# Montessori Preschool Elevates and Equalizes Child Outcomes: A Longitudinal Study

**DOI:** 10.3389/fpsyg.2017.01783

**Published:** 2017-10-30

**Authors:** Angeline S. Lillard, Megan J. Heise, Eve M. Richey, Xin Tong, Alyssa Hart, Paige M. Bray

**Affiliations:** ^1^Department of Psychology, University of Virginia, Charlottesville, VA, United States; ^2^Department of Education, University of Hartford, Hartford, CT, United States

**Keywords:** early childhood education, preschool, Montessori, cognitive development, social development, theory of mind, mastery orientation, academic achievement

## Abstract

Quality preschool programs that develop the whole child through age-appropriate socioemotional and cognitive skill-building hold promise for significantly improving child outcomes. However, preschool programs tend to either be teacher-led and didactic, or else to lack academic content. One preschool model that involves both child-directed, freely chosen activity and academic content is Montessori. Here we report a longitudinal study that took advantage of randomized lottery-based admission to two public Montessori magnet schools in a high-poverty American city. The final sample included 141 children, 70 in Montessori and 71 in other schools, most of whom were tested 4 times over 3 years, from the first semester to the end of preschool (ages 3–6), on a variety of cognitive and socio-emotional measures. Montessori preschool elevated children’s outcomes in several ways. Although not different at the first test point, over time the Montessori children fared better on measures of academic achievement, social understanding, and mastery orientation, and they also reported relatively more liking of scholastic tasks. They also scored higher on executive function when they were 4. In addition to elevating overall performance on these measures, Montessori preschool also equalized outcomes among subgroups that typically have unequal outcomes. First, the difference in academic achievement between lower income Montessori and higher income conventionally schooled children was smaller at each time point, and was not (statistically speaking) significantly different at the end of the study. Second, defying the typical finding that executive function predicts academic achievement, in Montessori classrooms children with lower executive function scored as well on academic achievement as those with higher executive function. This suggests that Montessori preschool has potential to elevate and equalize important outcomes, and a larger study of public Montessori preschools is warranted.

## Introduction

Optimizing preschool education is important from both economic and developmental standpoints (Heckman, 2006; Blair and Raver, 2016). The human brain undergoes marked development in the first 6 years, and the environment interacts with gene expression producing changes that appear to be permanent (Zhang and Meaney, 2010). Furthermore, neural development proceeds in a hierarchical fashion, with later attainments built on earlier ones (Merzenich, 2001). Economic analyses show that the highest rates of return on educational investments in human capital are derived from preschool programs (Heckman, 2006). Yet the two primary examples of successfull early childhood interventions (Perry Preschool and the Abecedarian Project) are from the 1960s (Campbell et al., 2002; Schweinhart et al., 2005) and were small studies with very intensive interventions that would be very expensive (on the order of $20,000/year per child) to implement in today’s dollars (Minervino and Pianta, 2014). Doing such interventions at scale would be exceedingly difficult. However, some alternative public preschool programs can feasibly be widely implemented; one such program is Montessori. Understanding if such programs provide measurable benefit to young children’s development is a prerequisite to determining whether to attempt implementation at scale.

Montessori education aligns with principles and practices that a century of research has shown are more optimal for child development than the principles and practices that undergird conventional schooling (Lillard, 2017). Developed by a physician in the first half of the 20th century, the educational method stemmed from close observation of children in relatively free environments. It provides a complex and interrelated set of hands-on materials and lessons across major topic areas and is designed for children ages 0 to 12+ years (Montessori, 1994a). Within a structure created by the materials and teacher oversight, children are free to make constructive choices among activities that they have been taught, to explore personal interests (with the caveat that they also engage broadly), and to decide whether to work alone or with peers in the multi-age classrooms. There are no grades or extrinsic rewards, and learning is situated in real or simulative contexts. Montessori education is aimed at development of the whole child, integrating social and cognitive growth for healthy independent functioning.

The first studies of Montessori outcomes lacked good controls or had small samples and compromises in program quality; for example, they used single-age classrooms, added non-Montessori activities, and/or had teachers with minimal training (Karnes et al., 1983; Miller and Bizzell, 1984). Program quality is clearly an important consideration, as children in higher-fidelity Montessori classrooms (where children had only Montessori activities) had larger social and cognitive school-year gains than those in lower-fidelity ones (Lillard, 2012). However, the Lillard () study had serious limitations, including that the children were middle-income and not randomly assigned to the schools, which were private. Such limitations are common in the relatively few existing studies of Montessori education (Rathunde and Csikszentmihalyi, 2005; Peng and Md-Yunus, 2014).

Another study avoided these problems by testing 5-year-olds in a high-fidelity public inner-city Montessori school who had gained admission through a computerized district-level random lottery when they were 3 years old, and compared their outcomes to those of 5-year-olds who had lost that lottery and were at non-Montessori schools (Lillard and Else-Quest, 2006). The Montessori children significantly outperformed the control children on an array of measures. In that study, however, the sample of preschoolers was small (*N* = 55), and the children were tested just once during the school year. These limitations are also problematic.

In the present study, children in two high-fidelity public Montessori magnet schools (11 classrooms) who had gained admission via a random computerized district-level lottery at 3 years old were compared to a group who had lost the lottery and attended other non-Montessori schools, over half of which were private schools. Children (*N* = 141) were tested over the fall semester when they were 3 years old, and then again at the end of the school year for three consecutive years. The tests, described next, assessed a variety of skills known to be important to later success.

Children’s academic ability is considered of primary importance in school assessments. For young children, initial progress in reading, vocabulary, and numerical understanding are valued indicators. Here we measured these with four Woodcock–Johnson IIIR Tests of Achievement: Letter-Word, Picture Vocabulary, Applied Problems, and Calculation (Woodcock et al., 2001). The Woodcock-Johnson tests have good psychometric properties as described in the manual, and are frequently used to measure school outcomes.

Academic benefit might have trade-offs in social learning; indeed, Montessori education has been criticized for being “asocial” since the children rarely participate in whole-class activities (DeVries and Gonçu, 1987). Social cognition was measured with the Theory of Mind scale (Wellman and Liu, 2004), which has good internal and external validity (Wellman, 2014); for example, it predicts later social competence (Wellman, 2014). A central construct in the Theory of Mind scale is understanding of false belief, which has garnered considerable attention in developmental psychology and education in the last 30 years (Blair and Razza, 2007). Understanding that someone can have a false belief entails the crucial understanding that minds represent the world, and that people’s behaviors are based not (necessarily) on the way the world actually is, but on how they represent the world to be (Dennett, 1987). The Theory of Mind scale contextualizes this key understanding with steps leading up to it (understanding of perception and its relation to knowledge, and understanding that people can believe different things) and following it (understanding that the emotions we convey might be different from the emotions we actually feel).

Although theory of mind is related to social competence, they are different constructs. Social competence was measured more directly with stories from the Rubin’s Social Problem-Solving Test - Revised (Rubin, 1988); a different story was used each year, and scoring was modified to home in on the maturity of social competence revealed in children’s responses. In these stories, one child has a coveted resource (like a swing) that another child really wants, and children need to come up with strategies the focal child could use to obtain the resource; responses like “I would ask her to share for 10 min then she could have it for 10 more minutes” are considered highly competent, whereas “I’d tell the teacher” or “I’d say please, please, please” are not. Other studies have shown that children in high-fidelity Montessori preschools show more social competence on this task (as well as better playground interactions) than children in other types of preschools (Lillard and Else-Quest, 2006; Lillard, 2012).

Theory of mind is also strongly associated with executive function and involves many of the same neural structures (for example the medial and lateral prefrontal cortex and the temporo-parietal junction) (Carlson and Moses, 2001; Koster-Hale and Saxe, 2013; Powell and Carey, 2017). Executive function was measured in this study because it undergirds self-regulatory skills that are important to academic and life success (Blair and Razza, 2007; Diamond, 2013; Vernon-Feagans et al., 2016); in fact, self-regulation at age 4 predicts health, wealth, and criminality outcomes at age 32 (Moffitt et al., 2011). Here executive function was measured with two tasks; a full battery of tests would have been desirable (Willoughby et al., 2011; Lipsey et al., 2017), but time constraints only allowed two. One executive function task was Head-Toes-Knees-Shoulders (HTKS), in which a child must do the opposite of a command (for example, touch their toes when asked to touch their head). To do this, a child must keep a command in mind along with the rule to execute its opposite, must inhibit the opposite response, and must executive the required one. This task has good psychometric properties and is related to other tests of executive function as well as concurrent and later academic success (McClelland et al., 2007; Ponitz et al., 2008, 2009; Lipsey et al., 2017). The second executive function assessment was the Copy Design subtest from the Visuospatial Processing section of the NEPSY-II (Korkman et al., 2007). For this task, children see a design, and must hold it in mind as they transform the visual image into its motor execution and a new resulting visual copy of that image. Thus working memory, attention, inhibitory control, and execution skills are employed. Design copy is highly related to other tests of executive function (Grissmer et al., 2010; Cameron et al., 2012; Fuhs et al., 2014; Lipsey et al., 2017) and has good test-retest reliability (*r* = 0.72 in Lipsey et al., 2017). Design copy ability is also related to academic achievement (Grissmer et al., 2010). Although both of these tasks require some similar executive function skills, HTKS involves large motor processes whereas Design Copy involves fine motor skills.

In addition to academic achievement, theory of mind, social competence, and executive function, which have been examined previously, we also used three tasks not previously used in studies of Montessori preschool. The first was the growth of a mastery orientation. Mastery orientation is an important personal quality (Dweck, 2006) indicative of a “growth mindset” (Dweck, 2017): a belief that with effort one can master challenges and increase one’s abilities. People who are mastery oriented want to learn, and take on challenging tasks in order to do so. They are resilient, persisting even in the face of failure. Their implicit theory of intelligence is that it is malleable, such that the harder one works, the better one can be. By contrast, people who are performance oriented seek to look good; their implicit theory of intelligence is that it is fixed, and they tend to give up in the face of failure. About 80% of Americans naturally adopt one orientation or the other, but circumstances can alter those orientations. Clearly if school could increase mastery orientation, this would be positive. Because conventional school practices like extrinsic rewards tend to instead encourage a performance orientation, and Montessori education does not use them, we expected that children might be more mastery oriented by the last 2 years of Montessori preschool. Mastery orientation was measured with a modification of a puzzle task developed by Smiley and Dweck (1994). Children were given an easy and a very difficult (actually, impossible) puzzle to solve, and then later were offered the opportunity to work on either puzzle again. Convergent evidence suggests that children who choose to continue to work on an unsolvable puzzle are “persisters” with a stronger mastery orientation than children who choose to work again on an easy puzzle (Smiley and Dweck, 1994). Having a mastery-oriented mindset predicts achievement over time (Dweck, 2006). Because it would take time for an orientation like this to develop in a school program, and because it involved a 0–1 response, choices at the first two vs. the last two time points were examined.

The second new construct was feelings about academic tasks. Early academic achievement might occur at the expense of enjoying school tasks, which is undesirable since enjoying kindergarten predicts later school achievement (Ladd et al., 2000). Not liking school tasks could stem from extensive emphasis on academics and could presage burnout, an issue recently raised with regard to a study of Tennessee preschoolers who performed less well by second grade than children who had not gone to preschool (Lipsey et al., 2015; Haskins and Brooks-Gunn, 2016). Therefore we assessed children’s liking of academic tasks such as school lessons and reading. However, because preschool-aged children tend to be very positive about many experiences, how much they professed to like leisure activities like playing and watching movies was also taken into account.

Another measure not used in prior studies of Montessori outcomes was the Alternate Uses task, which assesses creativity. Creativity is certainly a desirable construct. Because conventional educational methods often require children to answer questions in specific ways (as on multiple choice tests) but Montessori often encourages independent exploration, Montessori might promote more creativity. On the other hand, there are particular ways that children are instructed to use specific Montessori materials, and this could discourage creativity. Alternate Uses (sometimes called Creative or Unusual Uses) is a commonly used task that asks one to come up with as many uses as one can for common items like paper clips and towels (Guilford and Christensen, 1973). It was administered at each time point after the first fall. Many major current innovators, like both founders of Google (Sergei Brin and Larry Page), the founder of Amazon (Jeff Bezos), the creator of Wikipedia (Jimmy Wales) and the designer of the once-revolutionary video game Sim City (Will Wright) attended Montessori schools (McAfee, 2011; Gaylord, 2012), and other studies have shown that Montessori children are more creative in later grades (Lillard and Else-Quest, 2006; Besançon and Lubart, 2008), but not in preschool. To our knowledge, no other study has used Alternate Uses with Montessori preschool children.

In sum, the study measured children’s academic achievement, theory of mind and social skills, executive function, mastery orientation, relative enjoyment of school, and creativity at four time points to determine whether Montessori education would have a significant influence on those important constructs.

In addition to examining the overall efficacy of Montessori preschool for these measures, the study (because of its sample size) permitted examination of Montessori’s potential for disrupting the predictive power of certain variables for certain outcomes. One is the predictive power of income for achievement, or the income achievement gap. Childhood poverty is a significant predictor of poor life outcomes (Brooks-Gunn and Duncan, 1997; Yoshikawa et al., 2012). Education is widely viewed as a ladder out of poverty, yet socio-economic status (SES) and school achievement are correlated (National Early Childcare Research Network, 2005; Sirin, 2005). The income achievement gap, which is larger than the racial achievement gap, is present by kindergarten and persists at that high level throughout school (Reardon, 2011). Here we examined Montessori’s potential to address the income achievement gap in preschool. Second, executive function is known to predict many life outcomes (Moffitt et al., 2011); children with poorer executive function generally do not do as well in school (Blair and Razza, 2007; Duncan et al., 2007), and so remedial programs like the Chicago School Readiness Project (Raver et al., 2011) and Tools of the Mind (Diamond et al., 2007) are instituted as costly add-on programs. Montessori is a form of differentiated instruction that can naturally support different levels of executive function. For example, a child who needs more structure can be monitored more closely than a child who needs less structure. This is more difficult to do in conventional schools, since the structure is set up to treat all children in a given class in the same way (Tomlinson, 2014). Because Montessori can more easily and naturally accommodate differences in children, we ask whether executive function might be less predictive in Montessori programs.

The samples were ethnically diverse and equivalent at the first test point in terms of parent education and income (ranging from $0 to $200,000), child age, and Time 1 scores; this lack of pre-existing differences would be expected given the random lottery assignment. Slight (but non-significant) differences in performance at Time 1 could be due school programs already having influenced children at the first test point, which ranged from mid-September to mid-December. Over the subsequent 30 months, significant differences emerged on several measures, all indicating better outcomes for children in the Montessori program.

## Materials and Methods

This longitudinal study examined how children in Montessori vs. other preschool environments changed over 3 years. The same basic set of tests were administered to children at each time point. The study was carried out in accordance with the guidelines for human research of the Institutional Review Board for the Social and Behavioral Sciences at the University of Virginia, which approved the protocol.

### Participants

Sample characteristics are detailed in **Table 1**. In brief, the final sample included 70 children in Montessori and 71 controls who were at other non-Montessori schools. Children were 41.15 months old on average at the first test point, and each sample was ethnically diverse and had slightly more males than females. Household income ranged widely (because the lottery was for a magnet school) as did parent education; the average parent had some college education, but the range was from 9th grade through post-graduate. The two subsamples did not differ on any measured ethnographic variable.

**Table 1:** Sample characteristics.

	Montessori (*n* = 70)	Control (*n* = 71)
Age at Time 1	41.31 months	41.00 months
Gender	39 Male	38 Male
Household income	$73,208	$68,914
Income range	$0–180,000	$0–200,000
Mother education	6.70 (1.30)	6.64 (1.12)
Father education	6.37 (1.38)	6.13 (1.23)
**Ethnicity: subsample percentages**		
Caucasian	48%	37%
African–American	17%	15%
Hispanic	16%	23%
Asian	3%	4%
Multi-ethnic	16%	20%

*Age at subsequent test points: T2: +6 months; T3: +18 months; T4: +30 months. There were no significant group differences in any demographic variable, nor was there a difference in inter-test interval time, as indicated by *t*-tests. A Chi Square test revealed no significant sample differences in ethnicity (*p* = 0.63). Income: In both subsamples, 35% of sample < $50K household income; 80% < $100K. One family declined to state income. Parent Education: 1 = less than 9th grade, 2 = 9th grade, 3 = 10th grade; 4 = 11th grade, 5 = High School diploma, 6 = some College, 7 = College degree, and 8 = Post-graduate education*.

#### Recruitment

All participants were recruited from Hartford, CT and its outlying suburbs by letters sent home from the school district office following a school choice lottery (see below) in each of 4 years spanning 2010–2013; each participating child was in the study for 3 years, so data collection spanned from fall 2010 through spring 2016. Letters were sent to parents of all 3-year-olds who had been entered in a lottery listing one of two public Montessori magnet schools as their first choice; the letters were accompanied by contact, demographic, and school information forms, a permission letter, and an envelope to return their information to the study coordinator. Parents were sent a $10 gift card as a thank you for returning the information forms. After spring tests each year, children were sent an age-appropriate book and parents were sent a $50 gift card.

#### Lottery

The lottery was done by computer at the Connecticut State Department of Education’s Regional School Choice Office in Hartford, CT in May of each year. A child’s parent or guardian had submitted a lottery application during the period spanning October through February, selecting one of the two Montessori schools as their first of five school choices. The lottery selection was random except for neighborhood, sibling, and staff preferences. Staff children were disqualified from the study but 2 study children were admitted to a Montessori via the sibling preference; their siblings had presumably been admitted at random so the latent parent characteristics the lottery was intended to control for were still present. One control child had been admitted to Montessori but did not attend because the parents “did not like the neighborhood the school was in”; all other participants who gained admission to one of the two Montessori schools did become enrolled there. These two siblings and the admitted non-attender were assigned to the school program group they were actually in, but removing the two siblings and placing the cross-over child in the experimental group (“intent-to-treat”) had no meaningfully effect on results. For example, the ANCOVA on Time 4 academic achievement strengthens slightly when these changes are made, from *F*(2,119) = 7.24, *p* = 0.008, $\eta_{p}^{2}$ = 0.06 to *F*(2,117) = 9.58, *p* = 0.002, $\eta_{p}^{2}$ = 0.08. For philosophical reasons (such as grouping participants according to the treatment actually received) the study’s original group assignment was retained.

#### Schools

##### Control schools

Forty-three control children attended the same schools for the duration of their time in the study; 26 made one school switch, and 1 switched schools twice. At the beginning of the study, the 71 control children were in 51 schools; most of those schools had 1 child, some had 2–3, and one had 4. Over the course of the entire study (6 school years), control children were at 71 different schools. (Children were tracked at the school, not the classroom level). Thirty of the 71 schools were publicly funded (15 magnet including for example Reggio, Arts, and Environmental Science schools; 8 conventional public schools; and 7 Head Start programs) and 41 were private schools. Thirty-two of the schools attended by control children were in Hartford city (including West Hartford, which is wealthier with an average household income of $120,000) and 39 were in the outlying suburbs. Public early childhood programs in Connecticut must (1) satisfy the NAEYC accreditation standards and (2) be a member of the state’s early childhood professional registry. Connecticut requires an Early Childhood Teaching Credential that entails either (1) being a graduate of an approved higher education program or (2) another higher education degree, teaching experience, and 12 credits in early childhood education.

##### Montessori schools

One of the Montessori schools was the first public Montessori school in Connecticut, established in 1994. The other one opened in 2008. During the study years both Montessori schools were recognized by the Association Montessori Internationale (AMI) for their strict fidelity to original principles. One school had 5 classrooms and the other had 6 classrooms serving 27 three- to six-year-olds. One school also included students to 6th grade and the other to 8th grade; each had about 350 children in total. The teachers all had AMI training, for which a BA/BS degree is preferred but not required. Three of the teachers originally at one school had previously taught conventionally, and agreed to be retrained when the school converted to Montessori in 2008. There was some teacher turnover during the study but these changes were not tracked at either Montessori or conventional schools.

#### Missing Data and Exclusions

Over 4 years, 174 children were admitted to the study; 141 were retained in the final sample. Of these 141, 122 children were tested at all 4 time points, and 19 were tested at 3 time points. Of these 19, one joined the study at Time 2, 2 missed one test session, and 16 moved or crossed over between Time 3 and Time 4. 11 of these were in Montessori and 5 were control children. The control children who were lost had all moved; this lost subset of control children had performed significantly lower in academic achievement at earlier time points than the control children who did not move. The Montessori children who were lost at Time 4 did not significantly differ from those who remained in the study. Thus attrition patterns bias Time 4 results toward better outcomes for the control sample. For the variables reported here and the remaining children, 2.6% of data is missing due to experimenter error, child non-compliance, or interruptions in testing.

Of the 33 children who were admitted but excluded from the study, 23 children contributed insufficient data; 4 of these (2 Montessori) were lost between Times 1 and 2 and 19 (9 Montessori) were lost between Times 2 and 3. The children who were lost did not differ from other children in terms of parent education, parent income, ethnicity, or gender. The decision not to include these children was based on a preference for actual over imputed data. The other 10 excluded children (6 Montessori) had insufficient English (*n* = 5), speech delay (*n* = 3), or other learning disabilities (*n* = 2).

### Procedure

All parents provided written informed consent. Testing was conducted one-on-one, usually in the child’s school, but in a few cases in a public library due to lack of school cooperation. Ten trained research assistants tested children over the course of the study (eight graduate students and two project coordinators). Tasks were administered in a fixed order chosen to vary formats for engaging children: Theory of Mind, Letter-Word, Alternate Uses, Design Copy, Puzzle Part 1, Math, Head Toes Knees Shoulders, Social Problem-Solving, Picture Vocabulary, Preference Questionnaire, Puzzle Part 2. Testing was done simultaneously at Montessori and control schools so that test time would not be confounded with school type.

Participants were administered the same tasks at all test points, except the Preferences Questionnaire and the Alternate Uses creativity task, which were added in the spring of 2011, so these tasks are missing at Time 1 from the 29 participants who enrolled in 2010.

On some tasks, having exactly the same items at different test points would threaten validity. For these tasks there were four sets of materials, administered on a rotating basis.

#### Academic Ability

Children’s academic ability was assessed using the Woodcock–Johnson IIIR Tests of Achievement according to the instructions in the manual (Woodcock et al., 2001). Because there were no age differences across samples, raw scores were used for all Woodcock–Johnson tests. The Picture Vocabulary subtest assessed vocabulary, and the Letter-Word subtest assessed reading. Because the Montessori schools both taught cursive letters, the printed letters in the earlier items on the Letter-Word subscale were overlaid with cursive letters when testing Montessori students. Ordinary print letters were retained from the point when the test changes from letter to word identification. Early mathematical achievement was measured with the Applied Problems subtest, followed by the Calculation subtest if children scored 19 points or higher. These scores were summed for a Math score. The Math, Letter-Word, and Picture Vocabulary score loaded on a common factor (see **Appendix**) and were highly correlated (*r*s > 0.80), so to reduce the number of comparisons in the study, these scores were combined (by adding *Z*-scores) for an overall Academic Achievement measure (e.g., Lipsey et al., 2017).

#### Theory of Mind

We used four tasks from the Theory of Mind Scale (Wellman and Liu, 2004) omitting the lowest level (Diverse Desires) for brevity since 3-year-olds typically pass this level. As an example, in the Knowledge Access task, children were shown what was hidden in the drawer of a doll-house-sized bureau, and then shown a doll who they were told had not seen inside the drawer. They were asked if the doll knew what was inside the drawer, and if the doll had seen inside the drawer; both answers had to be correct for a child to be given credit. Children were given Knowledge Access first, followed by Contents False Belief, Diverse Beliefs, and Hidden Emotion, for final scores of 0–4. The contents, dolls, and doll names changed for each test session. For example, for contents false-belief task, one year the child saw a Band-Aid box with crayons inside, another year a raisin box with buttons inside, another year a Crayons box with rubber bands inside, and another year a Cheerios box with beads inside. Since children entered the study for four consecutive years, each material set came first for a portion of the sample.

#### Social Problem Solving

One object acquisition story from Rubin’s Social Problem-Solving Test - Revised was administered (Rubin, 1988) each year. In these stories, children were shown two other preschoolers, one of whom had a coveted resource like a swing and had had it for a “long, long time” and the other of whom wanted that resource. Children were asked what the second child could do or say to get the resource, what else they could do or say, and what the child him- or herself would do or say. Children’s use of strategies considering fairness and justice for both parties were coded. Although there is no limit to the number of such solutions a child might give, in reality the range was 0–3 at all four test points. Interrater reliability on 20% of all responses across all years was 0.99.

#### Executive Function

Executive Function was assessed with two tasks. For Head-Toes-Knees-Shoulders (Ponitz et al., 2009), children were first asked to touch their head, then to touch their toes. Children were then told that they were playing an “opposite game” in which they must touch the opposite part of the body than the experimenter said. Children were then administered 10 items, each scored 0–2, with 0 indicating the child followed the command literally, 1 meaning the child touched the incorrect body part first and then corrected themselves without prompting, and 2 meaning the child touched the correct (opposite) body part. If a child scored 10 points or more on the first 10 items, a second series of 10 items was administered which included knees and shoulders; the maximum points a child could earn was 40.

Second, the Design Copy subtest from the Visuospatial Processing section of the NEPSY-II was administered and scored according to the manual (Korkman et al., 2007). Children were shown a paper with a 4 × 4 grid with four figures across the top and third rows. The first figure was a vertical line; the experimenter showed children how to copy the line in the box below it (first box, second row), saying (for 3- and 4-year-olds), “See this line? I will draw one here. Now you draw one here,” handing the child the pencil and pointing to the second figure (a horizontal line) and the box below it. For 5-year-olds, and for the remaining items, the experimenter simply pointed to the top figure then the blank box below it, saying, “Copy this one here.” This continued for up to 16 figures until a child failed to successfully copy three figures consecutively. An independent coder coded a randomly selected subset of children at each test period, and interrater reliabilities across the two coders were excellent: *r*s = 0.98 (32 children at Time 1); 0.96 (22 children at Time 2); 0.95 (14 children at Time 3); 0.90 (22 children at Time 4).

#### Mastery Orientation

The puzzle task (modified from Smiley and Dweck, 1994) designed to test mastery orientation was given in two parts. First, children were given a fairly easy puzzle for their age, along with a picture of what the completed puzzle should look like. The picture was turned over while children solved the puzzle. After 2 min or when children completed the puzzle (whichever occurred first), they were given a much more difficult puzzle to solve and its completed picture which was then turned over. However, in this puzzle there were also pieces that had been switched with a similar puzzle, rendering the puzzle unsolvable. Children were again given 2 min to work on the puzzle. Then they completed several other tasks, and finally the experimenter brought out both puzzles again, told children that they had some extra time, and asked which one they wanted to work on and why; children could opt for neither or the easier puzzle (scored 0), or the more difficult puzzle (scored 1).

#### School Enjoyment: Preference Questionnaire

A questionnaire was developed to assess children’s enjoyment of academic (school and reading) and leisure (media and play) tasks; four filler questions were included as well. There were four questions about each of the focal topics, and children rated their enjoyment by pointing to a sad, neutral, or happy face. These responses were coded as 0, 1, or 2, and added together. Since young children often give the highest possible ratings on such scales (Ladd et al., 2000), to get variability, responses at the end of each school year (so they had experience with the school tasks) were summed, and liking for academic tasks was subtracted from liking of recreational tasks, reflecting how much more each child liked recreational than scholastic activities across preschool.

#### Creativity

Alternative Uses was used to assess creativity (Guilford and Christensen, 1973). First, as a warm-up, children were shown a photograph of an object (e.g., a pencil) and the experimenter said, “See this? This is a pencil. Can you tell me as many different things that you can think of that you can do, play or make with this?” If children made no reply in 10 s, the experimenter prompted with one use. The first of two test items was presented in the same way (“See this? This is a bucket…”). Responses were recorded for 1 min, with the experimenter prompting “What else?” If a child was producing responses and then appeared to run out of ideas (did not respond for a few seconds), the second item was shown and the same process repeated. For both test items the total time during which responses counted was 2 min; responses given after 2 min were not included.

Each intelligible response was scored as standard or non-standard. Categories were exclusive. For example, a standard use for a towel would be to wipe one’s body, and a non-standard use would be to place it over one’s head to pretend that one is a ghost. Analyses were conducted on the number of non-standard uses each child gave, collapsed across both items at each assessment. The actual range of responses was 0 to 5 total non-standard uses. Two coders independently coded a randomly selected subset of the data (*n*s below). Reliability was *r* = 0.80 on 16 children who were double-coded at Time 1; 0.73 (45 children at Time 2); 0.79 (46 children at Time 3); 0.82 (40 children at Time 4).

### Statistical Analyses

Some analyses reported here employed growth curve modeling, one of the most frequently used analytic techniques for longitudinal data analysis with repeated measures. Growth curve modeling can directly analyze intraindividual change over time and interindividual differences in intraindividual change (McArdle and Nesselroade, 2014). Growth curve analysis obtains a description of the mean growth in a population over a specific period of time. Individual variations around the mean growth curve are due to random effects and intraindividual measurement errors.

A typical growth curve model can be expressed as

$$y_{1}=\Lambda b_{i}+e_{i},$$

$$b_{i}=f(\beta ,X_{i})+u_{i},$$

where ***y***_i_ = (y_i1_,y_i2_,...,y_iT_)′ is a T × 1 vector and *y*_ij_ is an observation for individual *i* at time *j* (*i* = 1, ..., T; *j* = 1, ..., T where *N* is the sample size and *T* is the total number of measurement occasions); Λ is a *T* ×*q* factor loading matrix determining the shape of growth trajectories, ***b**_i_* is a *q* × 1 vector of random effects, and ***e**_i_* is a vector of intraindividual measurement errors. The vector of random effects ***b**_i_* varies for each individual, and its mean, representing the fixed effects, can be interpreted by a function of covariates ***X**_i_* with parameters β. The residual vector ***u**_i_* represents the random component of ***b**_i_*.

We use maximum likelihood estimation methods to fit the model. Missing values are believed to be missing completely at random (MCAR) or missing at random (MAR). Thus, Full Information Maximum Likelihood method (FIML) is applied to deal with missing data.

Data were not nested in control classrooms for the obvious reason that most control schools had only one child, and children’s classrooms and teachers were not tracked because they were not the focus of this study. Data were also not nested within Montessori classrooms, and the reason for this might be less obvious: Every year the 11 Montessori classrooms were differently constituted. First, peers changed: Always, at least 33% of children turned over as the oldest group of 9 moved on and a new group of 9 three-year-olds entered. In addition, several teachers and assistants turned over at some point during the study (although this was not closely tracked, at least three teachers at one school turned over), rendering different teacher experiences for each wave of children entering a given physical class (some had teacher A for 3 years, others for 2, others for 1, and others did not have teacher A at all). For this reason, treating children who entered a given classroom in 2010 and those who entered that classroom in 2013 as being in the same class (as a nested design would do) would not make sense; they had no overlap in peers, and many had different teachers as well. If we treated each entering year as different classrooms, we would have many tiny groups (1.6 children per nested group on average, given the average of 6.36 children per classroom entering over 4 years). Nesting Montessori children in classrooms therefore did not make sense. Analyses comparing results at the two Montessori schools revealed no school differences.

### Time 1 Equivalence

*T*-tests were done on all results to determine whether the samples differed already at their initial test (Time 1), conducted at some point during their first 3 months of school. The *p*-values exceeded 0.05 for all tests, indicating that the samples were equivalent at the start of the study.

The groups were slightly (although not significantly) different in academic achievement at the first test point. Since the children were randomly assigned to Montessori or the waitlist, it seems most likely that these small differences were due to their respective school programs beginning to have an effect between the time of school entry and the initial test point (which was mid-December for some children, 3 months into the school year). This is further supported by lack of group differences in all the demographic variables.

## Results

Here we first explain how data were reduced, then discuss the results showing that Montessori preschool elevated performance overall for the whole sample. We next discuss results showing that Montessori equalized performance of subgroups by raising the typically lower-performing subgroups towards the level of the higher-performing subgroups. We end with a comparison of public Montessori with public and private non-Montessori schools.

### Data Reduction

**FIGURE 1:**
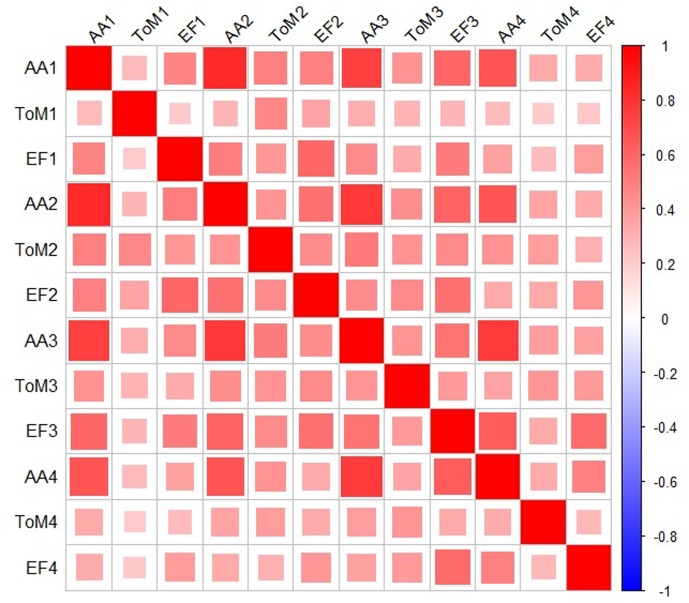
Correlation Table for Academic Achievement, Theory of Mind, and Executive Function across four time points. These variables were selected because their interrelations are of significant interest in preschool research. In this graphic representation, all squares are red because all correlations were positive. The shading legend is on the right. Darker colors (as well as larger squares) represent stronger correlations.

The Woodcock-Johnson scores loaded on a single factor and were significantly intercorrelated within each time point (*r*s > 0.80), so were converted to *Z*-scores and summed for an Academic Achievement score at each test point. The Copy Design and Head-Toes-Knees-Shoulders task also loaded on a single factor and were also significantly correlated (*r* = 0.66) so were converted to *Z*-scores and summed for each test point. **Figure 1** shows the correlations across the composite variables and Theory of Mind across time points, and the **Appendix** describes the factor analysis.

### Overall Findings: Montessori vs. Business-As-Usual

#### Academic Achievement

**FIGURE 2:**
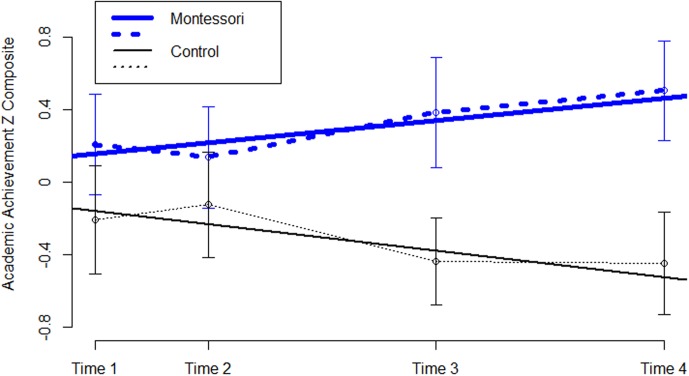
Academic achievement across preschool by school type. The figure shows significantly greater growth in academic achievement across preschool for children enrolled in Montessori preschool (dashed blue lines, *n* = 70) than waitlisted controls (dotted black lines, *n* = 71). Groups were statistically equivalent at Time 1 (the non-significant difference at Time 1 is likely due the Time 1 tests occurring into mid-December, thus school programs could already have made a difference) and Time 2 (late in the spring of their 1st year in preschool) and significantly different by the end of their 2nd and 3rd years in preschool (Times 3 and 4). Dashed/dotted lines represent actual data and solid lines represent fitted linear growth curves. Standard error bars are shown.

Although equal at the start of school, the Montessori group advanced at a higher rate across the study years, as illustrated in **Figure 2**; Δ*B* = 0.13 (*SE* = 0.067), *p* < 0.05. This initial analysis did not control for demographic variables because there were no differences, as would be expected given random assignment, but to confirm this a second growth model was created controlling for gender, household income, and Time 1 executive function. This confirmed that while both groups were equal at intercept in academic achievement, Montessori predicted a steeper slope of growth, whereas none of the control variables predicted a steeper slope in the overall sample. The result from the growth curve analysis was confirmed by an ANCOVA on Time 4 academic achievement, controlling for academic achievement at Time 1, *F*(2,119) = 7.24, *p* = 0.008, $\eta_{p}^{2}$ = 0.06. Independent samples *t*-tests showed that the groups were not yet different at Time 1 or Time 2, and that significant differences in academic achievement had emerged by the last two time points (approximately 4 and 5 years of age): *t*(136) = 2.10, *p* = 0.04, Cohen’s *d* = 0.36, and *t*(122) = 2.26, *p* = 0.03, Cohen’s *d* = 0.41, respectively.

#### Theory of Mind

Although children’s scores were equal at the initial test, a linear growth curve model showed that Montessori children had a significantly steeper rate of growth across the preschool years, Δ*B* = 0.10 (*SE* = 0.04), *p* < 0.05. This result remained in a second growth curve model that controlled for age, household income, and Time 1 Executive Function. Using a different analytic approach, an ANCOVA on Time 4 Theory of Mind scores controlling for Time 1 scores also showed a significant difference favoring the Montessori group, *F*(2,115) = 4.47 *p* = 0.04, $\eta_{p}^{2}$ = 0.04. Scores were examined at each time point. For Times 1 and 2 the two groups were not different. At Time 3, the difference was significant, *t*(135) = 2.09, *p* = 0.04, Cohen’s *d* = 0.36, and at the end of kindergarten (Time 4), the difference was a trend, *t*(122) = 1.74, *p* = 0.08, Cohen’s *d* = 0.32. These results show that social cognition developed more rapidly in children attending Montessori schools.

#### Social Problem Solving

Children in the two samples were equivalent throughout the study with respect to their social problem-solving skills; the average number of justice-related responses ranged from 0.24 to 0.97 across the 4 time points. An ANCOVA on Time 4 Social Problem Solving controlling for Time 1 comparing Montessori and control samples was non-significant *F*(1,117) = 0.20 *p* = 0.66, $\eta_{p}^{2}$ = 0.002, nor was the group difference significant at any time point with independent samples *t*-tests.

#### Executive Function

Linear growth curve analyses did not indicate differences in the growth of executive function. An ANCOVA on Time 4 executive function controlling for Time 1 only showed a trend toward a difference, with Montessori children scoring more highly: *F*(2,118) = 3.00, *p* = 0.09, $\eta_{p}^{2}$ = 0.03. Only at Time 3 was the difference significant, *t*(135) = 2.09, *p* = 0.04, Cohen’s *d* = 0.35. Evidence that Montessori magnet preschools lead to better executive function as compared to that developed by control children attending other preschools is not strong here.

#### Mastery Orientation

At the first two time points, there were no group differences: 37 of 70 Montessori (53%) and 35 of 71 control children (49%) chose to try a difficult puzzle again on one or both occasions (Fisher’s Exact test, *p* = 0.74). By the time children were 4 and 5, at Times 3 and 4, school program effects were significant, with Fisher’s Exact test showing more Montessori children made the mastery choice (45 of 69 or 65%) than did control children (33 of 71 or 47%), *p* = 0.03, two-tailed. Thus, children who were randomly assigned to a Montessori program were more likely to have a growth mindset by the latter half of their preschool years. Children’s explanations for their choices were consistent with the underlying orientation. Easy puzzle choosers said things like, “Because it’s easier,” whereas difficult puzzle choosers said things like, “Because I think I can do it.”

#### School Enjoyment

**FIGURE 3:**
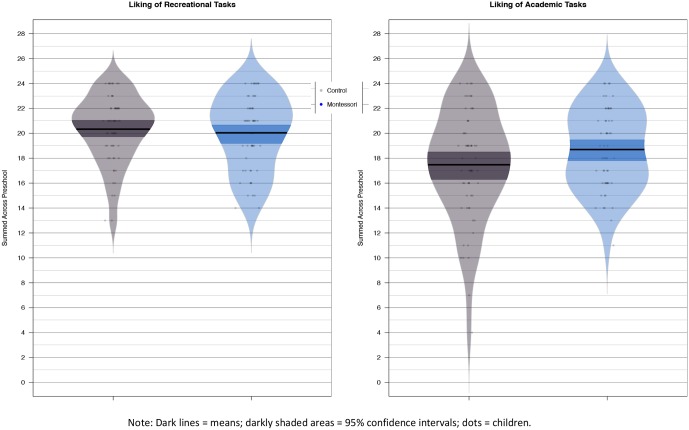
Enjoyment of recreational **(left panel)** and academic **(right panel)** activities across preschool. Montessori children (*n* = 55, blue beans, on right side of each panel) were relatively more favorable to academic tasks than control children (*n* = 63, gray beans). Dots represent children, bars represent means, and shaded areas represent 95% confidence intervals.

An ANOVA showed that the Montessori children were relatively more positive about school-related activities than were the control children, *F*(1,116) = 5.69 *p* = 0.02, $\eta_{p}^{2}$ = 0.05 (see **Figure 3**). This suggests that the Montessori children’s achievement gains were not at the expense of their enjoying school.

#### Creativity

Children in the two samples were equivalent throughout the study with respect to their creativity; average non-standard uses scores ranged from 0.31 to 1.55 across the 4 time points. An ANCOVA on Time 4 Creativity controlling for Time 1 Creativity comparing Montessori and control samples was non-significant *F*(1,94) = 0.96 *p* = 0.33, $\eta_{p}^{2}$ = 0.01, nor was the group difference significant at any time point with independent samples *t*-tests.

### Comparison of Subgroups in Montessori vs. Business-As-Usual Schools

We examined two sets of subgroups. First, we looked at the association of achievement with household income in Montessori vs. control schools. Because this achievement gap has been of considerable interest in the country historically, we present several analyses of this issue, before examining the influence of different levels of executive function in each sample.

#### Levels of Achievement for Children of Different Income Levels

**FIGURE 4:**
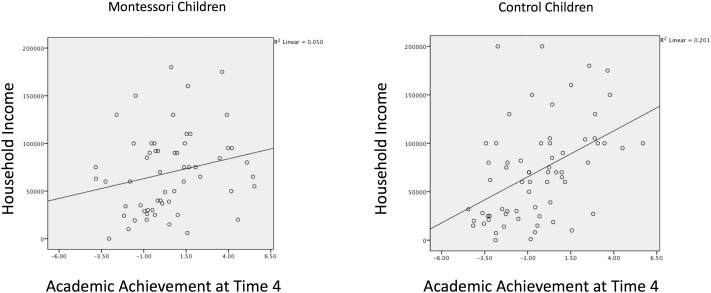
Relation between academic achievement and household income in Montessori and control children at the end of the kindergarten year. The relation is significantly smaller in Montessori children (*n* = 58, **left panel**) than in control children (*n* = 66, **right panel**).

Income is typically associated with school achievement. This was the case in the control sample, as shown in the right hand side of **Figure 4** using data from the final test point (Time 4). The left hand side shows this relation for the Montessori sample. Among children in Montessori, the correlation between academic achievement and household income across the entire study was 0.23, whereas in the control sample it was twice that: 0.46. Using the Fisher transformation, this difference in correlations was significant, *Z* = 2.46, *p* = 0.01. To further examine this, 1000 bootstrapped samples were generated; the 95% bootstrap confidence intervals of Δ*r* was (0.04, 0.39), supporting that the correlations between income and academic achievement in the two samples are significantly different. The smaller correlation among Montessori children might be a simple function of their being in magnet schools, since this is in essence the point of magnet schools [although their success at this is mixed (Ballou, 2009)]. However, for the subgroup of 15 control children who were at other magnet schools, the correlation between academic achievement and household income was even stronger, suggesting the mitigated income-achievement correlation for Montessori children is not merely due to their being in magnet schools.

**FIGURE 5:**
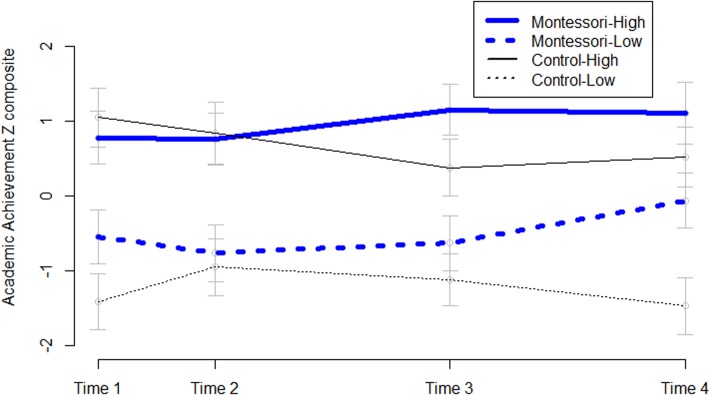
Academic achievement across four time points by school condition and income group. Although equal to the lower income control children at Time 1, by Time 4 the lower income children in Montessori showed a strong positive trajectory towards closing the achievement gap with the higher income children in control and Montessori schools. Standard error bars are shown.

How strong the gains in academic achievement were among just the lower income children is also of interest, because of the income achievement gap. Although the income range was very broad, there was not a sufficiently large subsample to only examine those living below the poverty line, so instead we examined the study subsample with a household income below the median split. For this lower income half of the sample (*n* = 67), mean household income was $32,627; *SD* = 18,443; the federal poverty line for a family of 4 in Connecticut was $24,600. At Time 1, an ANCOVA on academic achievement controlling for age (because there was a slight age difference in the subsamples), showed no difference between the Montessori and control lower income subsamples, whereas by Time 4 the lower income Montessori subsample had significantly higher academic achievement than the lower income control subsample, *F*(1,62) = 6.86, *p* = 0.01, $\eta_{p}^{2}$ = 0.10; see **Figure 5**. This result also held when controlling for Time 1 academic achievement: *F*(1,61) = 7.25, *p* = 0.009, $\eta_{p}^{2}$ = 0.11.

Furthermore, Montessori education greatly reduced the achievement gap across the preschool years. A series of four *t*-tests compared the lower income Montessori children with the higher income control children at each time point. For the higher income half of the sample (*n* = 74, including 7 at the median income of 70,000), mean household income was $105,804; *SD* = 33,123. The higher income control children outperformed lower income Montessori children at Times 1 and 2, *t*(64) = 2.47, *p* = 0.02, Cohen’s *d* = 0.61 and *t*(61) = 2.43, *p* = 0.02, Cohen’s *d* = 0.61, respectively. At Time 3, the difference was reduced by a third in terms of effect size and was no longer significant, *t*(62) = 1.59, *p* = 0.12, Cohen’s *d* = 0.40, and by the end of kindergarten (Time 4), the difference was reduced by yet another third, *t*(62) = 1.59, *p* = 0.41, Cohen’s *d* = 0.21. Thus, the effect size of the income achievement gap went from 3/5 of a standard deviation at age 3, to 2/5 at age 4, and finally to 1/5 at the end of the 3rd year in Montessori. Within the Montessori sample, the same series of tests showed trending (*p* = 0.06 at Time 1) or significant income-group differences in academic achievement at the first three time points but not at the last one, *t*(56) = 1.41, *p* = 0.16, although the difference was still a third of a standard deviation in size, Cohen’s *d* = 0.37. By contrast, within the control sample, the higher income subgroup performed a full standard deviation better than the lower income subgroup, Cohen’s *d* = 0.98. The higher income Montessori children were the highest performers in the study by the end of kindergarten (Time 4, see **Figure 5**), but the lower-income children were doing much better in Montessori classrooms than in control schools by this last time point.

#### Outcomes for Children with Different Levels of Executive Function

Second, we examined the predictive power of executive function for achievement. For both Montessori and control children, higher executive function predicted academic achievement at Time 1 (the intercept). In the control sample, as expected from many studies, executive function also predicted the slope of academic achievement in the latent growth curve model, Δ*B* = -0.067, *SE* = 0.03, *p* = 0.05. By contrast, initial levels of executive function had no influence on the slope of academic achievement for children in the Montessori programs, Δ*B* = 0.009, *SE* = 0.03, *p* = 0.76. Thus, in terms of academic outcomes, in Montessori classrooms children with low executive function do as well as children with high executive function. In other words, special supplementary curricula targeting executive function are not needed to equalize achievement outcomes for children in Montessori programs; academic achievement was higher overall, and children with lower executive function were not at a disadvantage.

### Montessori vs. Public or Private Business-As-Usual

Because control children were at both private and publically funded schools, we examined how Montessori children compared to both groups on academic achievement, theory of mind, and executive function. Controlling for academic achievement at the first time point, there was a significant school type effect on academic achievement at the final time point, *F*(2,122) = 3.94, *p* = 0.022, $\eta_{p}^{2}$ = 0.06. *Post hoc* tests showed a significant mean difference (favoring Montessori, for all results described here) between public Montessori and public control schools (*p* = 0.012) and a trend between public Montessori and private control schools (*p* = 0.055). There was no difference between public and private control schools (*p* = 0.42). For theory of mind, the same analyses indicated a group difference, *F*(2,114) = 4.30, *p* = 0.016, $\eta_{p}^{2}$ = 0.07, which *post hoc* tests revealed was both between public Montessori and public control schools (*p* = 0.004) and public and private control schools (favoring private, *p* = 0.048), but not between public Montessori and private control schools (*p* = 0.40). Executive function at the final time point controlling for the first time point approached a trend on the omnibus test *F*(2,117) = 2.27, *p* = 0.11, $\eta_{p}^{2}$ = 0.04 attributable to a significant difference in growth of children in public Montessori vs. in public control schools (*p* = 0.04).

## Discussion

Assisting young children’s development is an essential societal task; the human brain undergoes tremendous development in the early school years, setting in place patterns that predict life trajectories (Moffitt et al., 2011). Yet in the United States, the methods by which we try to help young children oscillate between didactic academic and pure discovery learning approaches, neither of which supports whole-child development in optimal ways (Fisher et al., 2011). Montessori education takes a different, whole-child approach and could feasibly be implemented at scale, but there have been no strong studies of its outcomes.

Taking advantage of a computerized random lottery for placement in two Montessori magnet preschools, this study compared 70 preschool-aged children who attended Montessori with 71 who did not. This is to our knowledge the first study spanning three years of Montessori education, and the second Montessori study to use a lottery-loser control design; the present study had a much larger sample size, and used new measures.

Montessori education elevated all children’s performance on several measures, and made the performance of groups that typically do less well more equal. First, academic performance of children in Montessori programs was significantly stronger over time. They performed slightly (but not significantly) better at the first time point, perhaps because children had on average almost 2 months of school program experience at the first test, with some children having a full 3.5 months. By the third and fourth time point, the differences in academic achievement were significant.

Furthermore, Montessori education made substantial headway in reducing the income gap in achievement across the preschool years. Whereas lower income control children were performing a full standard deviation lower than higher income control children by the end of preschool, the difference in income groups in Montessori was just a third of a standard deviation. Statistically, the lower income Montessori children did not differ from the higher income children in either school group by the fourth time point. In keeping with this, the income-achievement correlation was significantly smaller for children in Montessori than for children in the control group. This is a very important and impressive finding in our national search for ways to better help children born at an economic disadvantage.

Importantly, the higher achievement in Montessori was not at the expense of social skills or of liking school. Children who had by lottery ended up in Montessori programs performed better on tests of social cognition, were more mastery oriented, and expressed more liking of academic tasks relative to how much they liked recreational tasks. All these variables have predicted better outcomes in other studies, cited earlier. Montessori children fared equally well on tests of social problem solving and creativity, and had better executive function at age 4.

Finally, many studies have shown better academic and life outcomes for children with higher executive function or self-control. While for the control children in this study as well, executive function predicted academic achievement, this was not the case for children in Montessori. In Montessori classrooms, having lower or higher executive function did not matter for achievement; children with lower executive function performed as well as children with higher executive function in Montessori on academic achievement, which is impressive given that academic achievement in the Montessori sample was higher overall. Next we speculate on some possible reasons for these results, considering first intrinsic program differences in outcomes, followed by the possibility that Montessori teachers are superior.

### Academic Achievement

Children in Montessori programs excelled in academic achievement. The Montessori materials and presentations are one possible reason. The materials capitalize on the embodiment of cognition, for example having children trace letters as they say the letter sounds, and match cards with words to small objects. Ample research suggests that this is a more effective way to learn than sitting and listening (Lillard, 2017) as children often do in conventional preschool environments (Bassok et al., 2016). Furthermore, the content via which educational topics are approached in Montessori might be helpful. For example, in Montessori environments, children approach math through spatial learning, when Red Rods that systematically vary in length are transformed into Number Rods that name alternately colored segments with unit numbers (Montessori, 1914/1965, 1994b). The purpose of mathematics is to measure the physical world, and spatial and math skills are correlated (Verdine et al., 2017). Conventional education typically begins math education with counting discrete objects; perhaps starting with spatial relations as is done in Montessori is more helpful. In addition, the Montessori curricula and materials are very logical and very interesting (e.g., Montessori, 2016), and this could also be a reason for the difference. Another intrinsic program difference that could result in better learning outcomes is order. The Montessori environment and materials are also highly ordered, and more orderly environments are also associated with better cognitive and academic outcomes (Fisher et al., 2014). These are just a few of many possible reasons for the stronger academic outcomes for children in Montessori classrooms.

### Theory of Mind

This study aligns with two prior studies in showing that children in authentic (in this case, AMI-recognized) Montessori environments perform better on theory of mind than other children (Lillard and Else-Quest, 2006; Lillard, 2012). One possible reason for this is that Montessori classrooms combine children of three ages. In China, under the one-child policy, children in multi-age classrooms did better on theory of mind tests than children in single-age classrooms (Wang and Su, 2009). Other studies have shown that children with more older siblings also do better on theory of mind (Ruffman et al., 1998; Peterson, 2000). These advantages are believed to stem from the need to consider others’ mental states during conflicts that arise more often with similar-aged siblings or peers (Lillard and Eisen, 2017). A Montessori environment might present even more conflict than a typical preschool classroom, because there is only one of each type of Montessori material—one set of “Pink Tower” blocks, and one set of Musical Bells, for example. This scarcity in the context of 3-year age groupings might create challenges that lead to faster development in theory of mind. Alternatively, Dr. Montessori noted personality changes that accompanied deep concentration on work in preschool classrooms; one of these changes was to become more socially competent (Montessori, 1917/1965), which is associated with theory of mind; note, however, that the more direct measure of social competence (Social Problem Solving) did not show differences in this study.

### Mastery Orientation

Children in Montessori programs were more mastery oriented by ages 4 and 5 than were children in the control sample. One possible reason for this is the lack of extrinsic rewards in Montessori programs. The reward systems used in conventional school programs tend to lead to ability-oriented theories about oneself (Ames, 1992), which tend to go along with performance goals. People with performance goals tend to choose easier tasks that will make them look good (Dweck, 1999). Montessori programs encourage repetition of exercises to the point of mastery, and feedback comes from the materials rather than a teacher. These differences might explain the findings obtained here with regard to mastery orientation.

### Liking School Enjoyment

Although the children in this study all really liked recreational activities like watching television and movies and playing, children in Montessori showed relatively more liking of academic tasks like reading and getting lessons from a teacher. One possible reason for this is that children have choices about how they spent their time in Montessori; such choice is increasingly rare in preschool programs generally (Bassok et al., 2016). People are generally happier when they have choices, which provide a sense of self-determination (Deci and Ryan, 2011). Other possible reasons for more school liking dovetail with those given for achievement and mastery orientation.

### Executive Function

Unlike some other studies (Lillard and Else-Quest, 2006; Lillard, 2012; Kayılı, 2016), this study did not show significantly stronger development of executive function overall for children in Montessori; their executive function was significantly higher only at age 4. It might be that children whose parents enroll them in lottery magnets are different; this is the first study of magnet Montessori preschools. Alternatively, it might be that conventional preschools are improving in these areas because of social-emotional learning programs (Ursache et al., 2012). Further research is needed to tease apart these possibilities.

The finding concerning executive function and prediction of academic achievement is notable. Many studies have shown that executive function in the early school years predicts academic achievement (Blair and Razza, 2007; Duncan et al., 2007; Fuhs et al., 2014; Cameron et al., 2015), likely because in order for children to learn in conventional school they need to behave in ways that exercise executive function: They need to sit still, listen, follow directions, and inhibit engaging in other activities. But children across the full range of executive function who were in Montessori classrooms grew equally in academic achievement, and overall the Montessori children’s level of academic achievement was higher than that of controls. This suggests that having low executive function is not a disadvantage for children in this type of school program. Whether this translates to executive function being less predictive of later (such as Elementary school) outcomes for children who attended Montessori preschool is topic for further research.

One possible reason why executive function was not predictive of outcomes within the Montessori preschool program is that Montessori is a form of differentiated instruction. Children are not all treated alike; a child who needs more structure can be given that by the teacher. For example, a child who has not developed an ability to make constructive choices can be given limited, or even no choice, by the teacher, whereas a child who makes good choices (for example, chooses challenging work) is allowed to make their own choices. Closer examination of in-classroom processes, noting whether teachers do in fact scaffold lower executive function more effectively in Montessori programs, would shed light on this.

One might ask whether executive function near the time of school entry not predicting academic achievement is problematic. It does not seem so, since executive function still developed similarly in both groups and academic achievement was higher overall in the non-predictive group (Montessori).

### Montessori Teachers

In addition to intrinsic program differences, another possible reason for better Montessori outcomes is that Montessori teachers might be better teachers; if so, perhaps children in their classrooms would excel regardless of what educational program the teachers implemented. The teachers were not the focus of study here, but future research should consider this possibility. It is notable that at one of the two schools, three of six teachers had been teaching in a conventional way prior to 2008, and opted for retraining when the school adopted a Montessori program.

Considering the possibility that the study is revealing teacher rather than program effects, we note two points at which the Montessori teachers might have become better teachers: prior to their teacher training, or during (and as a result of) the teacher training.

#### Possible Pre-existing Differences in Teachers

First, one might ask whether the standards for entering a program to be a Montessori teacher are higher. Most of the Montessori teachers in this study trained at the AMI teacher training center in Hartford. Up to the time of this study, the training center courses were usually undersubscribed, so the center took virtually all applicants (Hall, personal communication, June, 2017). In addition, virtually all those who take the 9-month course are awarded a diploma. However, it is feasible that people who are attracted to Montessori teacher training interact differently with children, and this difference could be responsible for the results obtained. Other studies have shown non-trivial teacher effects at preschool. For example, a large study of prekindergarten classrooms in states that support pre-K (as does Connecticut) indicated two teacher variables that are most predictive of child achievement (Mashburn et al., 2008): (1) teacher emotional support, which predicts social outcomes and (2) teacher instructional support (asking high-level questions, scaffolding children’s thinking), which supports academic outcomes. It is possible that the Montessori teachers were higher on these variables even prior to their Montessori teacher training. Further research should examine this, perhaps through questionnaires given to people commencing Montessori vs. conventional teacher education programs.

#### Teacher Training Causing Teacher Differences

Second, the teacher training for Montessori might create better teachers. In terms of time and course intensity, the AMI training seems comparable to the training required for an early childhood teaching certificate. It involves 9 months of lectures and practice teaching, creation of a set of notes explaining Montessori theory and curriculum, and a final examination. The AMI “professors”—the people who teach the teacher-trainees—typically had at least 5 years as an AMI-certified classroom teacher followed by about 7 years of apprenticeship to another teacher trainer, so they are also highly trained. However, one difference to early childhood education is that in Montessori teacher training courses, one focuses on just one system and theory (Cossentino, 2005). By contrast, teachers in conventional teacher education programs typically learn many theories and methods. Whether learning a single theory or multiple ones creates better teachers is an empirical question.

**FIGURE 6:**
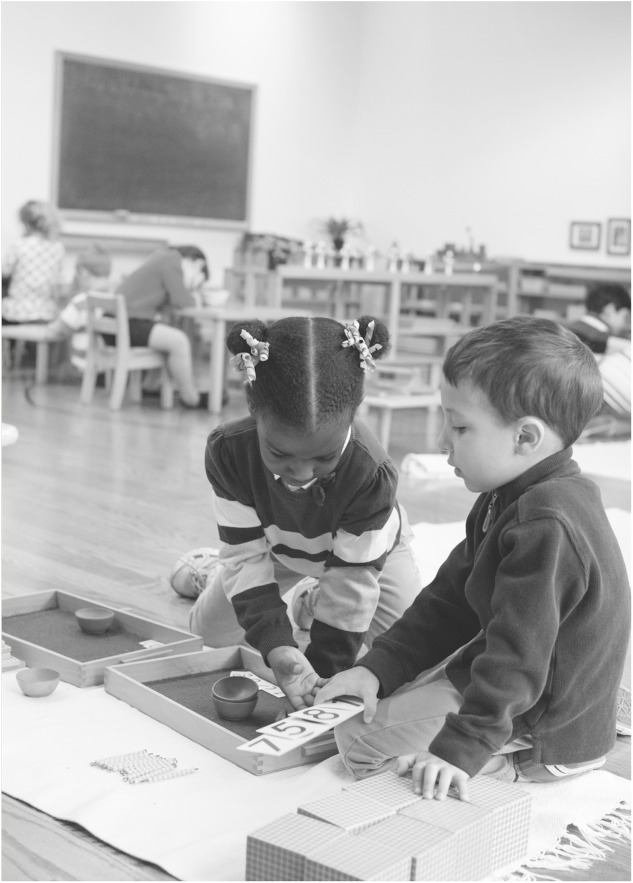
Two children working with Montessori decimal materials, with which preschool children perform multiplication and division of 4-digit numbers. Photograph by Laura Joyce-Hubbard, provided by courtesy of Forest Bluff School.

Another possibility, which also needs to be studied, is that Montessori teacher training changes teachers, perhaps by making them more sensitively responsive or higher in instructional support. If this is the case, then Montessori teachers are different but for a reason that is generic to Montessori education. Throughout Dr. Montessori’s books, a warm and loving attitude to children is expressed, and Montessori teachers are expected to come to embody this attitude (Lillard, 2017). In addition, Montessori teachers adopt high expectations of children, for example expecting them to achieve independence in ways that people rarely expect at least in American culture today. Even before age 3, Montessori children are expected to set the table, prepare a meal, and clean up, for example. Five-year-olds multiply and divide 4-digit numbers [see **Figure 6**; Montessori (2016) describes how this is achieved in high-fidelity Montessori classrooms], and carry out other complex tasks on their own. The combination of warmth, trust, and high expectations that is imparted to teachers during the Montessori teacher training might change them in ways that would make their students have better outcomes even if the teachers did not go on to implement a Montessori curriculum.

Various means should be used in future studies to look at the degree to which teachers might be responsible for better outcomes in Montessori education. First, one could examine attitudes toward and interactions with children prior to, during, and following teacher education courses, comparing those in Montessori and conventional training, to see how each type of teacher training changes people. Second, measures of teacher–child interaction could be used in studies like this, and entered as separate predictors in regression models, to see whether teacher interaction style in Montessori loads as or more strongly on outcomes than it does in studies of conventional teachers, for example using the CLASS (Pianta et al., 2012).

### Value-Added of Montessori Materials and Methods

Even if Montessori teachers differ in some ways from other teachers that cause better child outcomes, the Montessori materials and the methods with which the materials are used probably also add value. Two studies speak to this issue, both capitalizing on the fact that many Montessori classrooms do not offer exclusively Montessori materials. In one study, among 14 Montessori classrooms, children advanced more across a school year in classrooms that offered only Montessori materials than in “Montessori” classrooms that mixed in conventional materials like commercial puzzles (Lillard, 2012). In another study, conventional materials were removed midyear from two of three Montessori classrooms, and children in those two classrooms experienced significantly greater gains in the subsequent 4 months than children in the third classroom (Lillard and Heise, 2016). Because all the Montessori teachers in these studies were Montessori-trained, these studies suggest there might be something in the Montessori materials and the methods with which they are used that allow for steeper growth.

### Limitations

A major strength of this study is also a major limitation: It is based on a lottery for admission to two oversubscribed schools. Not all lottery entrants could be located (some had moved and left no forwarding address) and not all who were contacted agreed to enroll. School lottery entrants are not representative of all children, and oversubscribed schools differ from undersubscribed ones. In the real world, lottery designs are often the best available; longitudinal lottery studies are supreme. However, a lottery study is not as good as a true randomized control trial, where everyone is randomly assigned and is made to stay in their assigned group.

Another major strength that is also a limitation is that the study used high fidelity Montessori schools. Montessori outcomes appear to depend on the quality of the Montessori program (Lillard, 2012); outcomes at lower fidelity Montessori schools might not be the same. The Montessori programs in this study were recognized by the AMI, and we do not know if unrecognized Montessori schools, or ones associated with other Montessori organizations and teacher trainings, or even other AMI Montessori schools, would have similar outcomes. Another limitation is that the Montessori and control schools vary on many dimensions, and it is unclear whether specific dimensions might have contributed to outcomes, or whether Montessori programs must be fully implemented to have benefits. This study does suggest that very rigorous Montessori preschool programs significantly affect outcomes relative to business as usual, but less rigorous Montessori programs might not. Another limitation is that people who choose to become Montessori teachers might be different, and might teach more effectively regardless of program type. Ideally one could randomly assign future teachers to Montessori or conventional teacher training, but in lieu of that, other research strategies should be undertaken.

## Conclusions and Future Directions

Bearing these limitations in mind, the present study offers evidence that high fidelity Montessori preschool programs are more effective than other business-as-usual school programs at elevating the performance of all children, while also equalizing outcomes for subgroups of children who typically have worse outcomes. First, Montessori programs reduced the income achievement gap, raising achievement of lower income children well beyond the levels achieved by the lower income waitlisted controls. In addition, Montessori programs appeared to work as well for children who were lower in executive function at the outset as for children who were higher in executive function at the outset. Since preschool achievement predicts later achievement (Duncan et al., 2007), these benefits could feasibly extend upward, but whether they do so remains to be tested. Importantly these gains at preschool were not at the expense of “soft skills” that are the most important predictors of life outcomes (Heckman and Kautz, 2012).

Widespread implementation of Montessori programs would be premature prior to further research to examine the external validity of this study. There are over 450 public schools in the United States that offer Montessori education (National Center for Montessori in the Public Sector, 2014), and many of these admit by lottery. (There are also over 4000 private Montessori schools, but random lottery admission in those is unlikely). A large-scale study should examine outcomes in many more public Montessori schools, with an eye to Montessori implementation fidelity, as well as teachers and their training. The present study supports the legitimacy of such a study to determine more definitively if Montessori education should be implemented at scale.

## Ethics Statement

The study was carried out in accordance with the recommendations in the guidelines for human research of the Institutional Review Board for the Social and Behavioral Sciences at the University of Virginia, which approved the study protocol. Parents or guardians provided written consent for all children’s participation in accordance with the Declaration of Helsinki.

## Author Contributions

AL conceived of and obtained funding for the study, arranged with the sites, submitted initial IRBs, chose stimuli, oversaw all aspects of running, led effort in writing and statistical analyses and submissions. MH arranged for and did data collection in final study year, entered and cleaned data, maintained family contacts, assisted with analyses and writing. ER arranged for and did data collection in 5th year, entered data and maintained family contacts for 4 years. XT conducted growth curve and bootstrapping analyses as well as conceptualization of data, assisted with manuscript. AH created procedure manuals and materials sets, and maintained family contacts and data base, trained and maintained contacts with on-site RAs, and arranged for data collection visits in first several years of study. PB supervised RAs on site in Hartford, stored material sets, facilitated local contacts, provided Hartford school information, and assisted with manuscript.

## Appendix

The following factor model was fitted separately at each time point:

**Figure:**
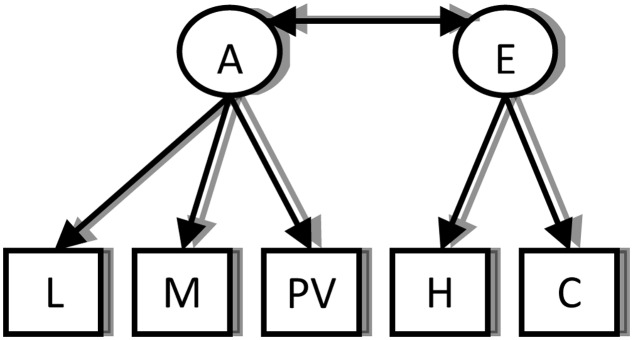


**Table A1** below shows the factor loadings and fit indices with factor loadings freely estimated. All models show excellent fit (from Kenney, 2015: for CFI, values over 0.9 are considered good; for RMSEA, 0.10 is the cut-off; for SRMR, less than 0.08 indicates good fit).

**Table A1:** Factor loadings and fit indices for academic achievement and executive function.

	Time 1	Time 2	Time 3	Time 4
**Academic achievement**				
Letter word	0.531	0.541	0.647	0.682
Math	0.945	0.906	0.785	0.789
Vocabulary	0.629	0.602	0.560	0.530
**Executive function**				
Head toes	0.182	0.639	0.531	0.612
Copy figures	0.198	0.547	0.498	0.540
**Fit indices**				
CFI	0.998	0.993	0.966	0.976
RMSEA	0.025	0.043	0.091	0.077
SRMR	0.030	0.029	0.034	0.031

A further analysis was done to determine fit with factors constrained to be equal; these results are shown in **Table A2**.

**Table A2:** Factor loadings and fit indices for academic achievement and executive function: constrained.

	Time 1	Time 2	Time 3	Time 4
**Academic achievement**				
Letter word	0.726	0.700	0.663	0.678
Math	0.726	0.700	0.663	0.678
Vocabulary	0.726	0.700	0.663	0.678
**Executive function**				
Head toes	0.195	0.588	0.512	0.575
Copy figures	0.195	0.588	0.512	0.575
**Fit indices**				
CFI	0.916	0.931	0.960	0.962
RMSEA	0.111	0.107	0.075	0.073
SRMR	0.072	0.071	0.050	0.058

In this analysis, for Time 1, when factors are constrained to be equal, model fit is more than adequate by two indices (CFI and SRMR) but by the RMSEA model fit is not good initially, when children are younger and there is more error (some very young children might not understand test instructions, for example); it becomes acceptable by Times 3 and 4.

## Conflict of Interest Statement

The authors declare that the research was conducted in the absence of any commercial or financial relationships that could be construed as a potential conflict of interest.
